# Research on Comparative Marine Atmospheric Corrosion Behavior of AZ31 Magnesium Alloy in South China Sea

**DOI:** 10.3390/ma18153585

**Published:** 2025-07-30

**Authors:** Tianlong Zhang, Shuai Wu, Hao Liu, Lihui Yang, Tianxing Chen, Xiutong Wang, Yantao Li

**Affiliations:** 1 State Key Laboratory of Advanced Marine Materials, Institute of Oceanology, Chinese Academy of Sciences, Qingdao 266071, China; 2 University of Chinese Academy of Sciences, Beijing 100049, China; 3 Southwest Institute of Technology and Engineering, Chongqing 400039, China; 4 College of Environment and Safety Engineering, Qingdao University of Science and Technology, Qingdao 266042, China

**Keywords:** marine atmospheric, magnesium alloy, field exposure, corrosion mechanism

## Abstract

In this study, the atmospheric corrosion behavior of AZ31 magnesium alloy exposed in Sanya and Nansha for one year was investigated. While existing studies have characterized marine corrosion of magnesium alloys, the synergistic corrosion mechanisms under extreme tropical marine conditions (simultaneous high Cl^−^, rainfall, and temperature fluctuations) remain poorly understood—particularly regarding dynamic corrosion–product evolution. The corrosion characteristics and behavior of AZ31 magnesium alloy exposed in Sanya and Nansha were evaluated using X-ray photoelectron spectroscopy, X-ray diffraction, electrochemical measurements, scanning electron microscopy, and weight loss tests. The results showed that the main components of corrosion products were MgCO_3_·xH_2_O(x = 3, 5), Mg_5_(CO_3_)_4_(OH)_2_·4H_2_O, Mg_2_Cl(OH)_3_·4H_2_O, and Mg(OH)_2_. The corrosion rate exposed in the Nansha was 26.5 μm·y^−1^, which was almost two times than that in Sanya. Localized corrosion is the typical corrosion characteristic of AZ31 magnesium alloy in this tropical marine atmosphere. This study exposes the dynamic crack–regeneration mechanism of corrosion products under high-Cl**^−^**-rainfall synergy. The corrosion types of AZ31 magnesium alloy in this tropical marine atmosphere were mainly represented by pitting corrosion and filamentous corrosion.

## 1. Introduction

The extensive adoption of magnesium alloys in critical industries such as aerospace, automotive manufacturing, consumer electronics, and medical implants is driven by their notable characteristics: low density, high strength-to-weight ratio, superior vibration damping, effective thermal dissipation, and inherent biocompatibility [1,2,3]. AZ31 magnesium alloys represent the optimal balance of cost, formability, and moderate corrosion resistance for marine applications like shipboard electronics housings and temporary marine structures. While alloys with higher aluminum content (e.g., AZ91) exhibit superior corrosion resistance, AZ31 dominates practical deployments where weight savings (density: 1.78 g/cm^3^ vs. AZ91 1.81 g/cm^3^), weldability, and extrudability are prioritized—making its degradation mechanisms in extreme tropical environments critically relevant [4,5,6].

The corrosion mechanism of magnesium alloys in the marine atmosphere is complex and affected by a variety of environmental factors. Under actual exposure conditions, the combined effects of temperature, relative humidity (RH), carbon dioxide concentration, chloride ion deposition rate, atmospheric particulate matter, and ultraviolet radiation can significantly accelerate the corrosion process of materials [7,8]. In general, the amount of chlorine-containing compounds in the atmosphere is low, and the harm to equipment in the environment is limited. However, in coastal areas, the decomposition of salt in seawater produces Na^+^ and Cl^−^, which can migrate through air currents and settle within a certain range, causing pollution and damage to the environment and materials [9,10].

High-humidity conditions are particularly prevalent in marine atmospheric environments, where magnesium alloys are more prone to severe localized corrosion in alternating humid and dry environments [11]. Cui et al. [12] showed that the corrosion rate of AZ31 magnesium alloy reached 17.66 μm·a^−1^ in the Xisha environment. Local temperature changes may alter the kinetics of chemical reactions due to the effects of heat conduction, resulting in differences between local metal temperatures and the overall average temperature [13]. In order to verify this phenomenon, some scholars have conducted indoor laboratories simulation experiments. Merino et al. [14] showed that the severity of corrosion increased significantly with increasing temperature and concluded that the order of corrosion intensity was AZ80 < AZ91D < AZ31 < Mg. The marine atmosphere is characterized by markedly greater chloride ion levels relative to inland or urban settings, a key factor driving its enhanced corrosivity. Liao et al. [15] conducted coastal environment experiments in Shimizu City and found that the corrosion rate was higher than that in the urban environment under the influence of sea salt, which explains the high corrosion rate. The dynamic characteristic of the marine atmosphere results in markedly elevated chloride deposition rates, typically ranging above 100 mg·m^−2^·d^−1^. Jiang et al. [16], utilizing the Scientific Research Vessel ‘KEXUE’, demonstrated that corrosion rates in the dynamic marine atmosphere exceeded those observed in static coastal exposures between five and eight times. The corrosion rate of AZ31 magnesium alloy was as high as 2.3 μm·a^−1^ in the steady-state chamber simulation in the atmosphere of 3% NaCl, which was much higher than the corrosion rate in field exposure test.

While previous studies have examined marine corrosion of magnesium alloys, the dynamic corrosion–product evolution mechanisms under extreme tropical marine conditions (simultaneous high Cl^−^ deposition > 400 mg·m^−2^·d^−1^, torrential rainfall > 2000 mm·y^−1^, and persistent high RH > 80%) remain unresolved. Specifically, the synergistic effects of rainfall-induced erosion and chloride penetration on corrosion–product layer stability require systematic investigation. Compared to field exposure, accelerated tests often fail to replicate synergistic environmental factors essential to tropical marine corrosion—particularly cyclic rainfall/UV-induced wet–dry transitions, atmospheric CO_2_ participation in carbonate formation, and real-time temperature/RH fluctuations.

The marine atmospheric environment of the South China Sea is characterized by high temperature, high humidity, and high Cl^−^; thus, the corrosion of metal materials is very serious in this situation [17,18]. In this study, the corrosion behavior of AZ31 magnesium alloy was investigated through a one-year field exposure testing in a tropical marine region. This study evaluated both the corrosion kinetics of AZ31 magnesium alloy and the structural/chemical properties of resulting corrosion layers. The degradation mechanisms governing AZ31 magnesium alloy corrosion in marine atmospheres were comprehensively elucidated.

## 2. Materials and Methods

### 2.1. Materials for Experiments

The material used was as-extruded AZ31 magnesium alloy (Shandong Hongtai Technology Co., Ltd., Zibo, China) in its industrial-delivery condition (no subsequent heat treatment). This represents typical as-fabricated components in marine applications. The magnesium alloy sample size is 100 mm × 50 mm × 4 mm. The density of AZ31 magnesium alloy samples is 1.78 g/cm^3^. The number of exposed samples per site is 20 pieces. The six sides of the magnesium alloy sample were polished sequentially, and the oil was ultrasonically removed in absolute ethanol. The sample was weighed with an analytical balance after drying, with an accuracy of ±0.0001 g, and the sample was marked. The samples were sanded and then polished with diamond paste before polishing.

### 2.2. Methods for Experiments

**Figure 1:**
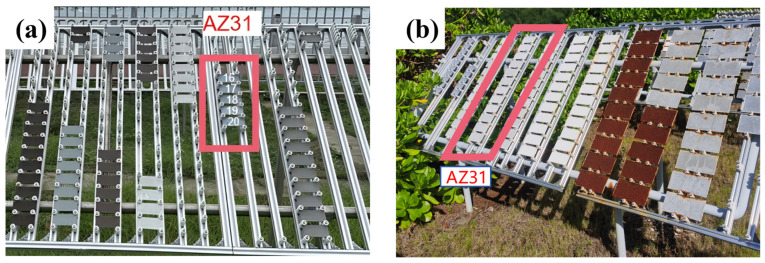
Samples of the extruded AZ31 magnesium alloy in Sanya (**a**) and Nansha (**b**) Marine Atmospheric Corrosion Test Stations.

The AZ31 magnesium alloy were placed on the racks of Sanya and Nansha test sites. The polymer material fixture was fixed on the experimental rack in turn, and each sample was spaced at a certain interval to prevent contact with each other. The placed sample was inclined at an angle of 45° to the ground, and the arrangement was shown in Figure 1. Five parallel samples were set up for each group of samples, and corrosion tests were carried out in the marine atmosphere environment in two places. Samples were taken after 1 year of cumulative exposure, and the retrieved samples were analyzed for weight loss, microscopic morphology characterization, and corrosion product analysis.

Next, 200 g/L CrO_3_ + 10 g/L AgNO_3_ solution was used to clean the corrosion products. The samples were soaked for 5 min in this solution, followed by sequential rinsing with deionized water and ethanol, concluding with cold air drying. The analytical balance was used to weigh the sample and calculate the weight loss of AZ31 magnesium alloy sample.

The corrosion rate of the sample is calculated using following formula:

(1) $$v\;={\;K\;\cdot (w}_{0}\;-{\;w}_{1})\;/\;(S\;\times \;T\;\times \;\rho )$$

where *w*_0_ and *w*_1_ represent the initial and final mass (g), respectively; *S* denotes the exposed surface area (m^2^); *T* is the exposure duration (days); *ρ* is the material density (g·cm^−3^); and *K* = 8.76 × 10^4^ is the unit conversion constant for mm·y^−1^ output.

Scanning electron microscopy (sigma500, Zeiss, Oberkochen, Germany) was used to characterize the microscopic morphology of magnesium alloy samples after environmental exposure tests, and X-ray diffraction (XRD) (PANalytical X-pert 3, Panaco, Almelo, The Netherlands) and X-ray photoelectron spectroscopy (XPS) (Nexsa, Thermo Scientific, Houston, TX, USA) were used to analyze the composition of corrosion products.

## 3. Results and Discussion

### 3.1. Macroscopic Corrosion Morphology and Environmental Impact Analysis

**Figure 2:**
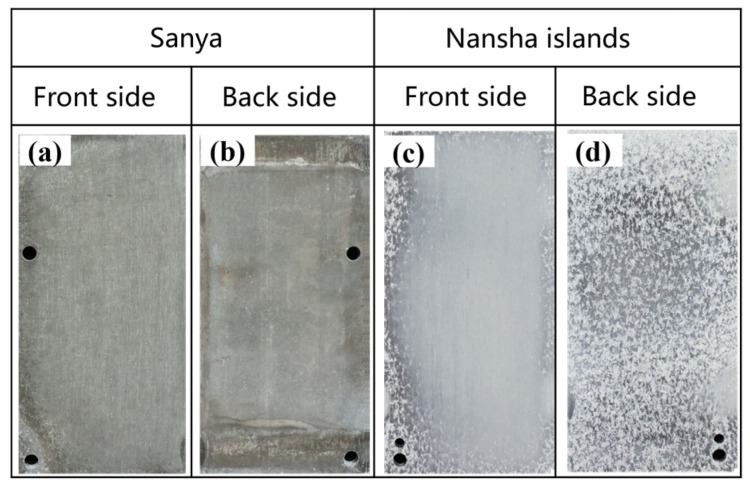
Macroscopic morphology of AZ31 magnesium alloy exposed for 1 year in Sanya and Nansha. Sanya (**a**,**b**) and Nansha (**c**,**d**).

Figure 2 shows the macroscopic morphology of AZ31 magnesium alloy exposed to the South China Sea for one year, and the surface of the alloy has lost its metallic luster. The surface of the AZ31 magnesium alloy exposed to Sanya is fully covered by dark gray corrosion products (Figure 2a), and the corrosion product film is relatively intact. While the back edge is darker (Figure 2b), the corrosion product coverage is incomplete. AZ31 magnesium alloy exposed to the Nansha is covered by silvery-white corrosion products (Figure 2c). The edge and reverse side (Figure 2d) show obvious pitting craters and are covered with gray and white speckled corrosion products. The pits on the back are relatively dense and the macroscopic morphology is uneven, which is quite different from the morphology of AZ31 in Sanya.

The tropical marine atmospheric environment of Sanya and the Nansha in the South China Sea are characterized by high relative humidity (RH), high temperature, and intense solar radiation. Under the influence of these environmental factors, the surface of the magnesium alloy sample exposed in Sanya and Nansha undergoes a periodic cycle of alternating wet and dry. Both regions exhibit comparable levels of key environment factors, specifically average temperature, average RH, and total solar radiation intensity. While the annual rainfall in the Nansha can reach 2078 mm, the chloride deposition rate is as high as 400 mg·m^−2^·d^−1^ (significantly higher than that of Sanya). Chloride ions, which are the main corrosion factor, can cause serious corrosion problems for equipment [19].

**Figure 3:**
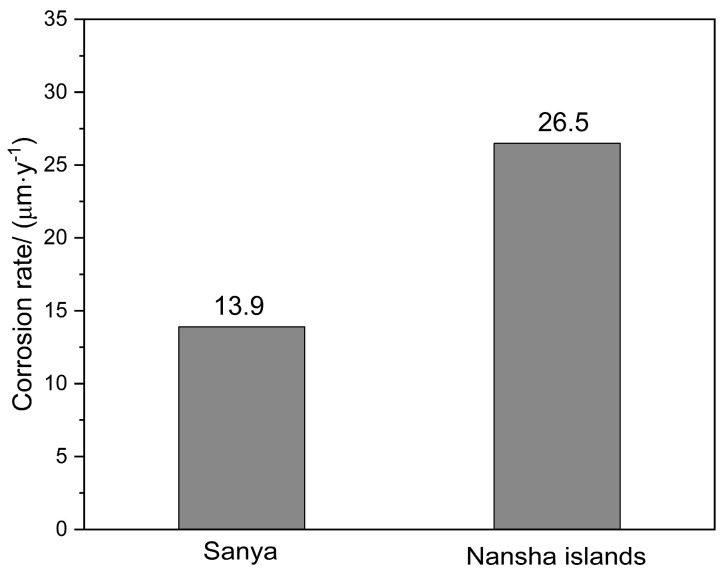
Corrosion rate of AZ31 magnesium alloy after one-year exposure (mean ± SD, *n* = 3). Nansha: 26.5 ± 1.8 μm·y^−1^, Sanya: 13.9 ± 1.2 μm·y^−1^ (*p* = 0.0003). Error bars denote 95% confidence intervals.

Figure 3 shows the corrosion rate of AZ31 magnesium alloy after one year of exposure at two sites. The corrosion rate of AZ31 magnesium alloy in Sanya is 13.9 μm·y^−1^, while that of AZ31 magnesium alloy in Nansha is 26.5 μm·y^−1^, which is significantly higher than that in Sanya. Compared with AZ91 magnesium alloy with the same exposure conditions and exposure period, AZ31 magnesium alloy corrodes faster and more severely. This divergence is primarily driven by unique environmental regime, characterized by intensive rainfall cycles and extremely high chloride deposition rates in Nansha. AZ31 magnesium alloy contains 3 wt% Al, while AZ91 magnesium alloy has 9 wt% Al. This higher Al concentration in AZ91 promotes a greater volume fraction of cathodic β-phase (Mg_17_Al_12_), intensifying micro-galvanic coupling with the α-Mg matrix.

Notably, trace impurities also contribute to cathodic activation: Fe (0.003 wt%) and Cu (0.003 wt%) in AZ31 (Table 1) form highly efficient cathodic sites despite their low concentrations. As demonstrated by Chen et al. [20], Fe impurities >0.005 wt% can dramatically accelerate corrosion rates by facilitating hydrogen evolution. While in the samples of this study, Fe content (0.003 wt%) remains below this critical threshold, its synergistic effect with Cu (which lowers the hydrogen overpotential) still enhances cathodic activity—particularly in chloride-rich environments like Nansha where these micro-cathodes sustain localized corrosion [21]. This impurity effect compounds the primary vulnerability from lower β-phase fraction of AZ31 relative to AZ91.

**Figure 4:**
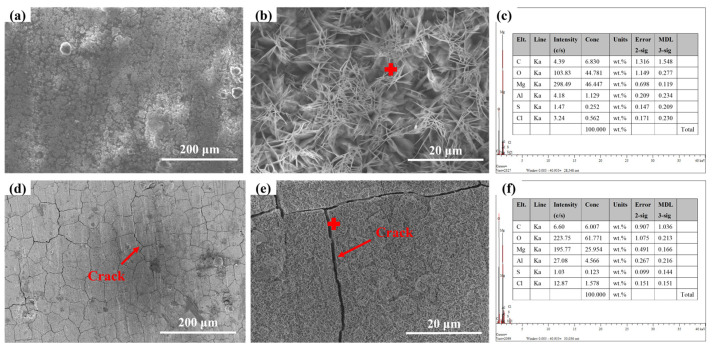
Microstructure of AZ31 magnesium alloy exposed for one year at the experimental sites in Sanya (**a**,**b**) and Nansha (**d**,**e**); (**c**,**f**) are the EDS of Sanya and Nansha, respectively.

Figure 4 shows the microscopic morphology of AZ31 magnesium alloy exposed in Sanya and Nansha. The results showed that the AZ31 magnesium alloy (Figure 4a) exposed in Sanya is covered by floral and cellular corrosion products, and the coverage of the matrix is uneven. At larger magnifications, the corrosion products appear as acicular clusters (Figure 4b). The microscopic morphology of the corrosion products (Figure 4c) of the Nansha is sheet-like and cell-like, and obvious cracks can be observed, indicating poor protection of the corrosion product layer. Meantime, vertical streaks were observed in the corrosion product layer.

The rainfall of the Nansha is abundant, and the vertical stripes of the AZ31 magnesium alloy corrosion products in this area may be related to rainwater erosion. The microscopic morphology at high magnifications showed that the corrosion products appeared to be flaky, with significant cracks present and had healed. This result may be related to the high humidity, high temperature, and high radiation in the Nansha, which makes the surface of magnesium alloy undergo a process of alternating dry and wet. Cracks within the corrosion product layer enable chloride ion (Cl^−^) infiltration to the substrate interface, triggering localized precipitation of secondary corrosion products (e.g., Mg(OH)_2_, Mg_5_(CO_3_)_4_(OH)_2_·4H_2_O) that seal microfractures. This autogenous healing process progressively increases corrosion product thickness through repetitive dissolution-reprecipitation cycles.

**Figure 5:**
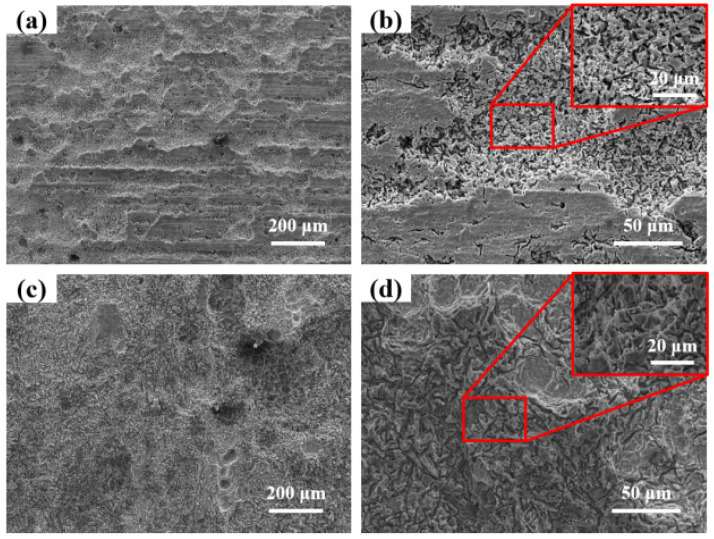
Microscopic morphology of AZ31 magnesium alloy after one-year exposure in Sanya (**a**,**b**) and Nansha (**c**,**d**) (Surface corrosion products have been cleaned up).

Figure 5a shows the microscopic morphology of the corrosion products of AZ31 magnesium alloy after one year of exposure in Sanya, and a considerable number of local corrosion pits and non-corroded matrix can be observed. As can be observed in Figure 5b, the gullies in the corrosion pit are crevice corrosion, which is the main form of localized corrosion. Figure 5c shows the corrosion morphology of AZ31 magnesium alloy exposed to the Nansha, with most of the surface corroded, indicating that the high chloride deposition rate and high rainfall in the region accelerated the corrosion of AZ31 magnesium alloy. From Figure 5d, the gullies and circular pits are observed, and the corrosion type is consistent with the AZ31 magnesium alloy pattern in Sanya, which is crevice corrosion.

**Figure 6:**
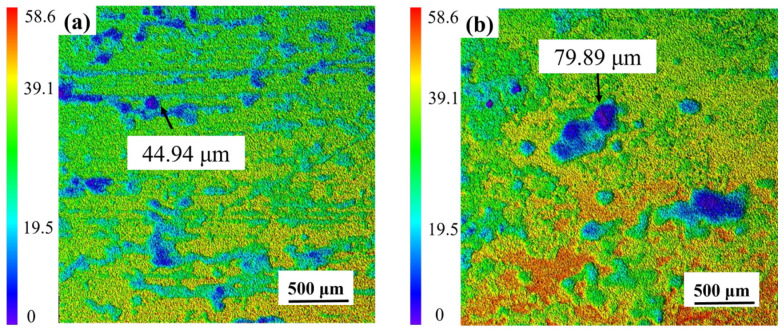
Laser confocal microscopy analysis of AZ31 magnesium alloy after one-year exposure in Sanya (**a**) and Nansha (**b**) (Surface corrosion products have been cleaned up).

Laser confocal micrographs in Figure 6a characterize corrosion-induced surface degradation on AZ31 after the one-year field exposure in Sanya tropical marine environment. Three-dimensional corrosion topography shows many small, shallow corrosion pits, while the uncorroded areas are relatively intact, with a maximum corrosion depth of 44.94 μm for the entire area. Figure 6b presents the 3D surface topography of AZ31 magnesium alloy following the one-year marine atmospheric exposure in Sanya, acquired via laser confocal microscopy. The three-dimensional corrosion morphology is uneven, accompanied by large and deep corrosion pits, which is significantly different from the corrosion depth of AZ31 magnesium alloy exposed to Sanya, and its maximum corrosion depth is 79.89 μm, consistent with the weight loss results (Figure 3). The corrosion difference between the two regions is also shown in Figure 2. As can be seen from Figure 2, the corrosion in the Nansha area is obviously more serious than that in Sanya. Both Sanya area (Figure 2a) and Nansha area (Figure 2b) show that the backside corrosion is more serious than the front side, and the front edge part is more serious than the central part.

**Figure 7:**
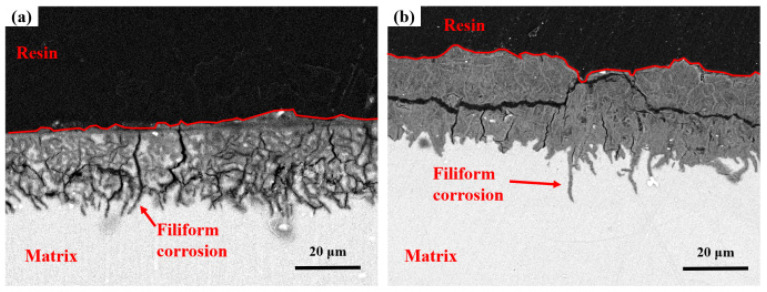
Cross-section microscopic morphology at center regions of AZ31 magnesium alloy after one-year exposure in Sanya (**a**) and Nansha (**b**).

Cross-sectional analysis in Figure 7a characterizes corrosion product stratification on AZ31 after the one-year tropical marine environment field exposure in Sanya. The gray area in the corrosion product layer is corroded matrix with many small cracks and partial penetration into the matrix. At the junction between the corrosion product layer and the matrix, it can be clearly observed that the crevice corrosion develops into the matrix. Figure 6b shows the microscopic cross-sectional morphology of AZ31 magnesium alloy exposed for one year in the Nansha, and the corrosion depth is much higher than that of AZ31 magnesium alloy exposed in Sanya, which is consistent with the weight loss results (Figure 3). The corrosion product layer is denser than that of the magnesium alloy in Sanya, but there are also many tiny cracks, in which corroded matrix particles are found. The corrosion type of AZ31 magnesium alloy exposed in the Nansha is crevice corrosion, which develops into the interior of the matrix in the direction of depth.

This crevice-dominated morphology contrasts with classical chloride-induced pitting expectations due to three synergistic factors: ① Dynamic corrosion–product fracturing: high rainfall (2078 mm·y^−1^) generates hydraulic stress on corrosion layers, creating micro-crevices at defect sites (Figure 4c) that evolve into macro-crevices (Figure 5d). ② Differential aeration enhancement: crevice geometries stabilize oxygen concentration gradients more effectively than pits under cyclic wet–dry conditions (RH > 80%), accelerating interior acidification. ③ β-phase distribution: the elongated grain structure of extruded AZ31 aligns intermetallic particles along extrusion directions, promoting linear crevice propagation rather than isotropic pitting.

While chloride ions (400 mg·m^−2^·d^−1^) initiate both corrosion types, the extreme environmental dynamics in Nansha favor crevice progression through (i) continuous electrolyte replenishment in crevices during rainfall, and (ii) UV-induced polymer degradation of temporary protective films within crevices [22]. This explains why observations in this study differ from other marine studies [23], where stable chloride layers promote pitting corrosion.

**Figure 8:**
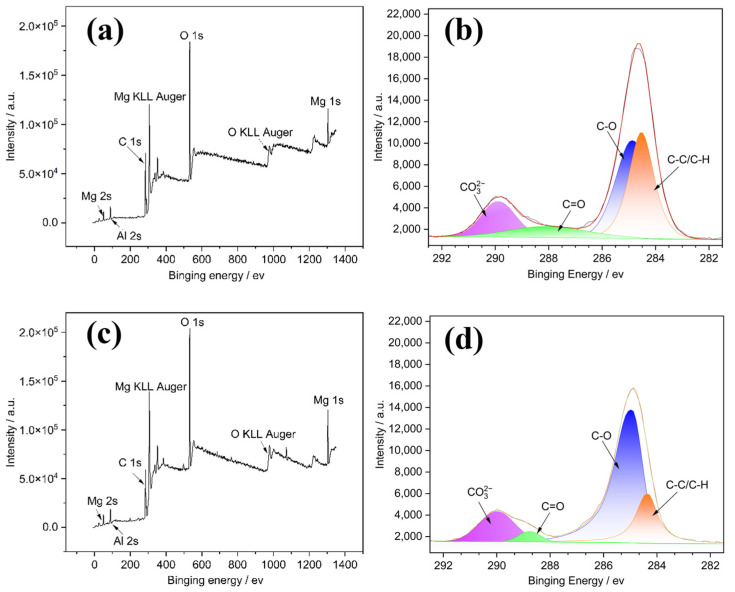
XPS analysis of the corrosion products formed on the surface of AZ31 magnesium alloy: (**a**) and (**c**), respectively, represent the whole spectra of Sanya and Nansha; (**b**) and (**d**), respectively, represent the C 1s spectrum of Sanya and Nansha.

Figure 8a,c shows the whole range of binding energy survey of Sanya and Nansha, which shows that there is no significant difference between Sanya and Nansha. Both samples are predominantly composed of O, Mg, C, and a trace amount of Al. Figure 8b,d illustrate a comparison of the high-resolution C 1s XPS spectra of the outer corrosion products. High-resolution C 1s spectra of the outer corrosion products (Figure 8b,d) were divided into two distinct components. The first component, centered at 285.9eV, is attributed to carbon species in C-C, C-H, and C-O groups. The second peak, observed at 290.5 eV, corresponds to carbonate (CO_3_^2−^) species. This indicates a significant presence of carbonate-containing compounds, such as Mg_5_(CO_3_)_4_(OH)_2_·4H_2_O, within the surface layer.

**Figure 9:**
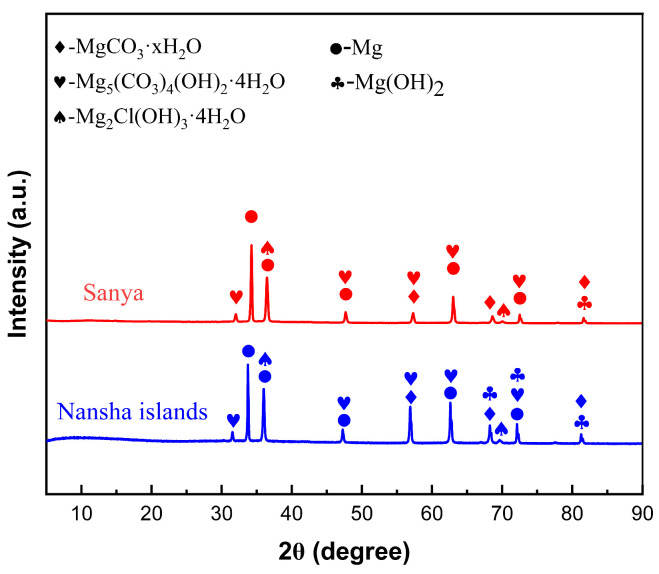
XRD spectrum of corrosion products of AZ31 magnesium alloy exposed for one year in Sanya and Nansha test sites.

Figure 9 shows the XRD analysis of AZ31 magnesium alloy exposed for one year in Sanya and Nansha. The corrosion product composition of AZ31 magnesium alloy exposed for one year in Sanya and Nansha was analyzed. The results showed that there was no significant difference in the composition of the corrosion products of AZ31 magnesium alloy exposed to these two places, and the main products were MgCO_3_·xH_2_O(x = 3, 5), Mg_2_Cl(OH)_3_·4H_2_O [24,25,26,27], Mg_5_(CO_3_)_4_(OH)_2_·4H_2_O, and Mg(OH)_2_.

**Figure 10:**
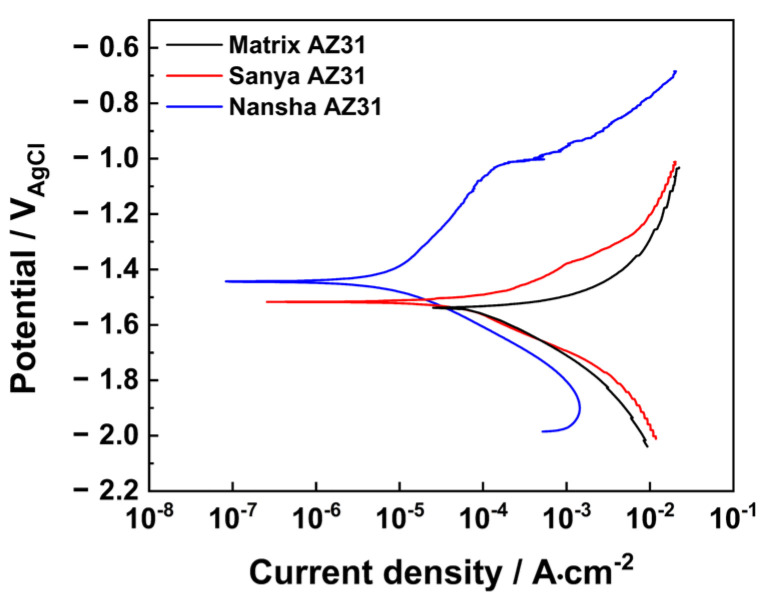
Dynamic potential polarization curve of AZ31 magnesium alloy exposed for one year in. Sanya and Nansha.

Figure 10 shows the Tafel polarization curves of AZ31 magnesium alloy exposed for one year in Sanya and Nansha. The results in Table 2 show that the corrosion potential of AZ31 was positively shifted compared with the matrix during the one-year exposure period in Sanya, and the corrosion current density decreased by 50.7%. The corrosion potential of AZ31 magnesium alloy exposed in the Nansha is further positively shifted compared with that of Sanya magnesium alloy, and the corrosion current density is reduced by 91.5% compared with that of the matrix, which is an order of magnitude lower than that of the magnesium alloy in Sanya.

### 3.2. Corrosion Mechanism

Based on the research findings, the significant corrosion divergence between the Nansha and Sanya arises from the synergistic amplification of extreme environmental factors unique to the Nansha. Critically, the Nansha exhibit an exceptionally high chloride deposition rate of 400 mg·m^−2^·d^−1^—substantially exceeding levels in Sanya—coupled with torrential annual rainfall reaching 2078 mm. This combination drastically accelerates electrolyte formation on the alloy surface during wet cycles. Dissolved Cl^−^ readily penetrates defects in the corrosion product layer, initiating severe pitting at cathodic β-Mg_17_Al_12_ phases. Concurrently, intense rainfall physically erodes corrosion products, generating vertical cracks that expose fresh substrate. Crucially, sustained high temperature and humidity in the Nansha prolong wet–dry alternation frequency. This dynamic environment promotes continuous “fracture-regeneration” cycles. XRD/XPS-identified compounds play distinct mechanistic roles: Initial cracks are sealed by rapid formation of hygroscopic Mg_2_Cl(OH)_3_·4H_2_O (chloride phase, Figure 9), which subsequently transforms into stable MgCO_3_·xH_2_O and Mg_5_(CO_3_)_4_(OH)_2_·4H_2_O through atmospheric CO_2_ incorporation (XPS C 1s at 290.5 eV, Figure 8b,d). New Mg-carbonates/hydroxides (MgCO_3_·xH_2_O, Mg_2_Cl(OH)_3_·4H_2_O) rapidly form within cracks, creating a thicker but more fractured barrier layer. Electrochemical tests confirm this regenerated layer reduces overall anodic activity, yet the maximum pit depth nearly doubles (79.89 μm vs. Sanya 44.94 μm) due to sustained Cl^−^ ingress through micro-cracks. In contrast, milder chloride exposure (lower deposition rate) and reduced rainfall in Sanya limit both electrolyte aggressivity and regeneration-driven cracking, resulting in shallower degradation.

When the magnesium alloy is exposed to corrosive medium, pitting corrosion and filamentous corrosion occur due to the exposure of the β-Mg_17_Al_12_ phase distributed along the surface grain boundary. The β-Mg_17_Al_12_ phase acts as the cathode and electrochemically reacts with the magnesium matrix that acts as the anode. Simultaneously, corrosion propagation advances through both intergranular and trans-granular pathways, accelerating penetration depth. Structural failure of the corrosion product layer—attributable to hygrothermal stress cycling (ΔT > 15 °C/day, RH fluctuations > 60%) induced by intense UV radiation and diurnal environmental variations—compromises protective functionality. This process exacerbates interfacial decohesion between the α-Mg matrix and cathodic β-Mg_17_Al_12_ phase, establishing preferential galvanic pathways that elevate corrosion kinetics. Following film rupture [28,29], the ingress of Cl^−^ and O_2_ facilitates reparative reactions at the substrate interface. Newly formed corrosion products occlude fractures, increasing film thickness by 15–30% per cycle while partially restoring barrier properties. This autogenous repair mechanism initiates a cyclical sequence of fracture–densification–regeneration: Crack infilling by secondary precipitates (primarily Mg(OH)_2_/MgO) enhances local compactness, though accumulated internal stresses propagate subsurface microcracks that perpetuate degradation.

### 3.3. Comparative Analysis with Literature

The corrosion rate of AZ31 in Nansha (26.5 μm·y^−1^) significantly exceeds values reported in other marine environments.

This divergence highlights the synergistic amplification unique to tropical dynamic marine environments. Unlike laboratory observations of Merino, where AZ31 corrosion decreased with Al content, field data of this study show β-phase (Mg_17_Al_12_) distribution dominates localized corrosion under high Cl^−^/rainfall—explaining higher vulnerability of AZ31 than AZ91 despite lower Al content (3 wt% vs. 9 wt%).

Crucially, the observed ‘crack–regeneration’ mechanism resolves a key contradiction in the literature: while electrochemical tests show reduced average corrosion current (Table 3), the maximum pit depth nearly doubles in Nansha. This reconciles model [30] of corrosion–product protection with observation of Feliu of “accelerated localized degradation under cyclic wet-dry conditions.”

## 4. Conclusions

The corrosion behavior of AZ31 magnesium alloy exposed to the tropical marine atmosphere of the South China Sea (Sanya and Nansha) for one year was systematically investigated. The main conclusions are as follows:

(1) AZ31 magnesium alloy exhibits severe localized corrosion in tropical marine atmospheres, primarily manifested as pitting corrosion and crevice corrosion. Filamentous corrosion features were observed in microscopic morphologies, consistent with prior studies. This localized degradation poses critical risks to structural integrity and service life.
(2) The corrosion rate in the Nansha was nearly twice that in Sanya. This divergence is attributed to the significantly higher chloride deposition rate and extreme annual rainfall in the Nansha, which intensified electrolyte formation and accelerated electrochemical reactions. The synergistic effect of rainfall-induced erosion and chloride infiltration on the corrosion product layer is the reason for the severe corrosion of the AZ31 magnesium alloy in this area.
(3) This study resolves the knowledge gap regarding corrosion–product evolution under extreme tropical conditions by demonstrating how high Cl^−^ flux (>400 mg·m^−2^·d^−1^) synergizes with torrential rainfall (>2000 mm·y^−1^) to drive a unique ‘crack–regeneration’ process. Electrochemical tests revealed that the samples exposed in Nansha exhibited lower corrosion current density and positive shift in corrosion potential, indicating a denser but dynamically regenerated corrosion product layer. Corrosion products in both regions comprised MgCO_3_·xH_2_O(x = 3, 5), Mg_2_Cl(OH)_3_·4H_2_O, Mg_5_(CO_3_)_4_(OH)_2_·4H_2_O, and Mg(OH)_2_.
(4) Based on the ‘crack–regeneration’ mechanism and chloride-driven degradation, AZ31 magnesium alloy corrosion resistance in tropical marine environments can be enhanced through hydrophobic coatings to disrupt wet–dry cycles; β-phase distribution control via thermo-mechanical processing to mitigate micro-galvanic corrosion; and rare-earth alloying (Ce/Gd) to form chloride-penetration-resistant inter-metallics.

## Figures and Tables

**Table 1:** Chemical composition of magnesium alloy.

Element (wt%)	Al	Zn	Mn	Si	Fe	Cu	Ni	Mg
AZ31	2.93	0.68	0.25	0.02	0.003	0.003	0.0006	Bal.

**Table 3:** Chloride deposition rates and rainfall in different regions.

Location	Corrosion Rate (μm·y^−1^)	Cl^−^ Deposition (mg·m^−2^·d^−1^)	Rainfall (mm·y^−1^)
Nansha	26.5	400	2078
Sanya	13.9	35	1200
Xisha [31]	17.66	64.39	1536
Shimizu [32]	9.8	4.2	2300
Lab-simulated [33]	2.3	-	-

**Table 2:** Tafel parameters of AZ31 magnesium alloy dynamic potential polarization curve exposed for 1 year in Sanya and Nansha.

Location	Self-Corrosive Potential (vs. SCE)/V	Corrosion Current Density (μA cm^−2^)	*b*_c_ (mV dec^−1^)
Unexposed AZ31	−1.5313	93.28	174
Sanya AZ31	−1.5172	45.95	140
NanshaAZ31	−1.4330	7.90	158

## Data Availability

The raw/processed data required to reproduce these findings cannot be shared at this time as the data also forms part of an ongoing study.
